# Draft genome of *Paraburkholderia fungorum* sequence type 868 recovered from human synovial tissues

**DOI:** 10.1016/j.dib.2019.104159

**Published:** 2019-06-20

**Authors:** Shih Keng Loong, Kim-Kee Tan, Nurul-Izzani Zulkifle, Sazaly AbuBakar

**Affiliations:** a Tropical Infectious Diseases Research & Education Centre, Faculty of Medicine, University of Malaya, 50603 Kuala Lumpur, Malaysia; b Department of Medical Microbiology, Faculty of Medicine, University of Malaya, 50603 Kuala Lumpur, Malaysia

**Keywords:** Emerging infection, Environment, Infectious disease, Malaysia, Sequencing

## Abstract

*Paraburkholderia fungorum* is an opportunistic bacteria infrequently associated with human infections. Here, we report the draft genome sequence of *P. fungorum* strain BF370, recovered from the synovial tissue of a patient in Malaysia. The *P. fungorum* genome contains a 8,950,957 bp chromosome with a G+C content of 61.8%. Colicin and heavy metal resistant genes were also present in the genome. Conserved sequence indels unique to *P. fungorum* were observed in the genome. The draft genome was deposited at the European Nucleotide Archive under the sample accession number ERS1776561 and study accession number PRJEB17921.

**Table:** Specifications table

Subject area	*Genetics, Genomics and Molecular Biology*
More specific subject area	*Microbiology*
Type of data	*Figure, tables*
How data was acquired	*Ion Personal Genome Machine System*
Data format	*Raw and analyzed*
Experimental factors	*Genomic DNA from bacterial pure culture*
Experimental features	*Isolation of bacteria, genome sequencing, draft genome assembly and annotation*
Data source location	*Kuala Lumpur, Malaysia*
Data accessibility	*Data have been deposited in public repository.* The draft genome was deposited at the European Nucleotide Archive under the sample accession number ERS1776561 (https://www.ebi.ac.uk/ena/data/view/ERS1776561) and study accession number PRJEB17921 (https://www.ebi.ac.uk/ena/data/view/PRJEB17921).

**Table:** 

**Value of the data** • The draft genome sequences add to the limited number of P. fungorum genomes available in the public databases. • Data can be compared with other P. fungorum genomes to provide insights into the possible mechanisms of pathogenesis. • Data may help in understanding the strategies used by P. fungorum to infect human and evade detection by the immune system.

## 1 Data

**Fig. 1:**
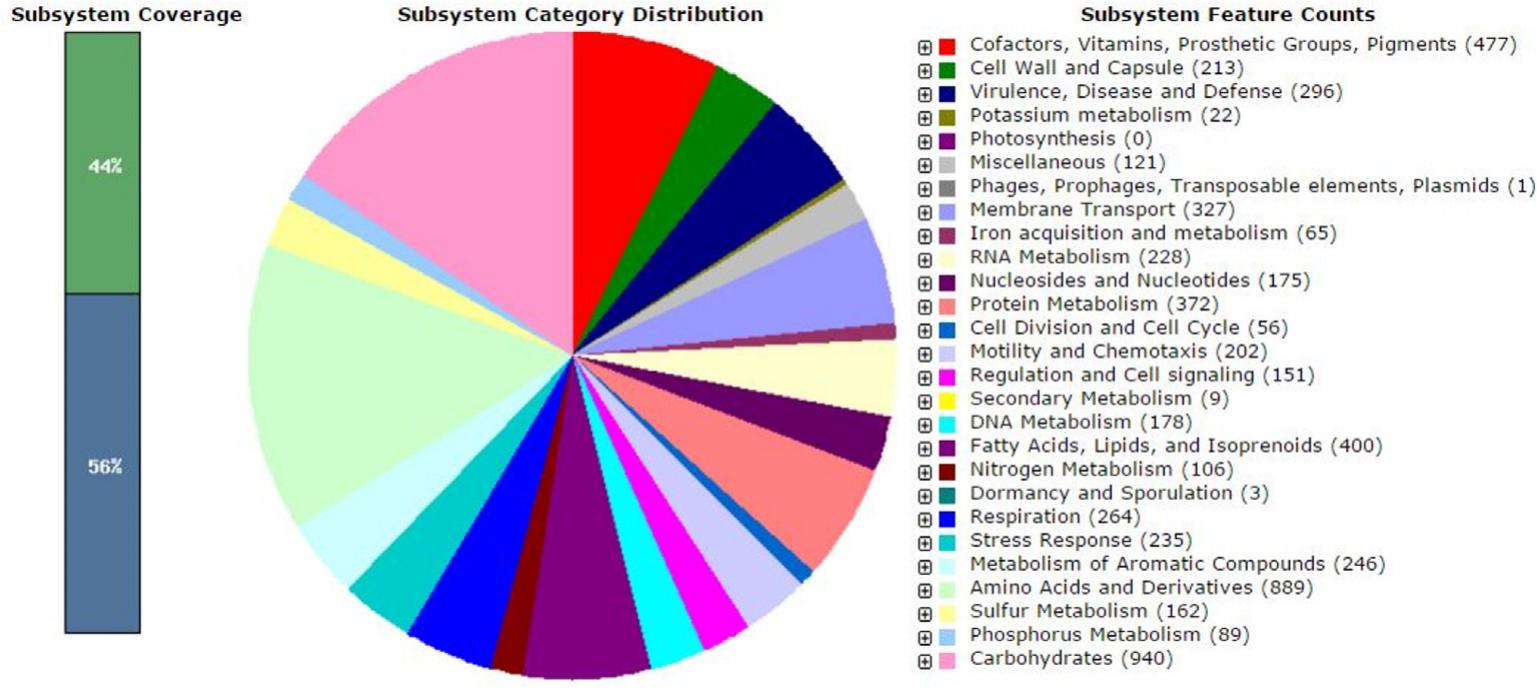
An overview of the subsystem categories assigned to the genome of *Paraburkholderia fungorum* strain BF370.

The complete genome of *P. fungorum* strain BF370 was 8,950,957 bp in length, comprising of 161 contigs with N50 of 119,394 (Table 1). The G+C content of the genome sequence was approximately 61.8% (Table 1). Nine copies of rRNA were predicted in the genome by using the RNAmmer V1.2 while 57 copies of tRNA were predicted by using IonGAP (Table 1). A total of 9,715 protein-coding genes were predicted using Rapid Annotation using Subsystem Technology (RAST), with 44.0% of the proteins included in the subsystem (Fig. 1). Amino acid sequence analyses revealed that *P. fungorum* strain BF370 contained the following conserved sequence indels (CSI); transposase A-like protein, group 1 glycosyl transferase and undecaprenyl-phosphate glucose phosphotransferase. These are molecular signatures unique to the genus *Paraburkholderia* [1]. *P. fungorum* is the only member of this environment-associated bacterial genus capable of causing human infections [1], [2], [3], albeit rarely. A novel sequence type 868 (ST868) was obtained upon sequencing and analyses of the seven housekeeping genes according to the multi locus sequence typing scheme for *Burkholderia cepacia* complex (https://pubmlst.org/bcc).

**Table 1:** General genome features of *Paraburkholderia fungorum* strain BF370.

Attribute	Value
Genome size (bp)	8,950,957
G+C content (%)	61.8
Contigs	161
ORFs	9715
rRNA genes	9
tRNA genes	57

Antimicrobial resistance genes against the beta lactams (beta lactamase Class A, Class C, metal-dependent hydrolases of the beta-lactamase superfamily I and metallo-beta-lactamase superfamily protein) were found in the genome, alongside genes encoding for extracellular antibacterial colicin and other bacteriocins. RAST server also uncovered the presence of genes capable of hydrolyzing heavy metals including, arsenic (*ArsH*), chromium (*ChrA*), copper (*CcmF*, *CcmH*, *CopC* and *CopR*), cobalt-zinc-cadmium (*CusA*, *CzcA*, *CzcB*, *CzcC* and *CzcD*) and mercury (*MerT*). Another defining feature of *P. fungorum* BF370 was the identification of beta-lactamase proteins in its genome, some of them not found in *P. fungorum* strains NBRC102489 and GAS106B (Table 2).

**Table 2:** Comparison of the presence of beta lactamase proteins in the genomes of different *Paraburkholderia fungorum* strains.

No.	Subsystem	Role	Strain (Study accession no., source)		
			BF370 (PRJEB17921, human synovial tissues)	NBRC102489 (PRJDB250, *Phanerochaete chrysosporium*)	GAS106B (PRJNA331287, soil)
1	Beta lactamase	Beta-lactamase (EC 3.5.2.6) - Class C	+	+	–
2		Beta-lactamase (EC 3.5.2.6)- Class A	+	+	–
3		Metal-dependent hydrolases of the beta-lactamase superfamily I	+	–	–
4		Metallo-beta-lactamase superfamily protein	+	–	–

+, present; -, absent.

## 2 Experimental design, materials and methods

Bacterial genomic DNA was extracted using Nucleospin Tissue kit (Macherey-Nagel, Düren, Germany) and, the purity and quantity were determined using NanoDrop 3300 Fluorospectrometer (Thermo Scientific, Wilmington, DE, USA). The whole genome sequencing of the *P. fungorum* was performed as previously described [4]. Briefly, the genome library preparation was carried out by using the Ion Xpress™ Plus Fragment Library Kit (Thermo Fisher Scientific, USA). Genome libraries corresponded to 200 bp sequencing were prepared using E-Gel^®^ SizeSelect™ Agarose Gel, 2% (Thermo Fisher Scientific, USA). The sequencing template was prepared using Ion OneTouch™ 200 Template Kit V2 DL (Thermo Fisher Scientific, USA) according to the manufacturers protocol. Amplified Ion Sphere Particles were enriched using IonPGM Enrichment beads (Thermo Fisher Scientific, USA). Genome sequencing was undertaken using the Ion Personal Genome Machine System (Life Technologies, USA). The low quality reads at the 3′-end regions and adaptor sequences (P1) were trimmed using default parameter as implemented the Torrent Suite V5.0.0. There were 1,304,128 reads with mean read length of 178 bp. Among the reads, 202,720,603 bp were ≥ Q20 bases. The pre-processed Ion Torrent reads were assembled *de novo* using SPAdes V3.1.0, using uniform coverage which was suitable for the sample with GC (35–68%) as implemented in Torrent Suite. The assembled genome sequences were uploaded to IonGAP for the prediction of putative tRNA and to RNAmmer v1.2 server for the prediction of putative rRNA. The assembled contigs were functionally annotated with RAST following the default RASTtk pipeline parameter.
